# Development and Validation of a Novel Circadian Rhythm-Related Signature to Predict the Prognosis of the Patients with Hepatocellular Carcinoma

**DOI:** 10.1155/2022/4263261

**Published:** 2022-08-12

**Authors:** Yumeng Wu, Cheng Shen, Xinghui Wang, Wenjing Zhao, Yuanyuan Li, Xiao He, Yuanbin Chen, Jibin Liu, Xuming Wu, Aiguo Shen

**Affiliations:** ^1^Cancer Research Center Nantong, Affiliated Tumor Hospital of Nantong University, China; ^2^Department of Computer Science and Engineering, Tandon School of Engineering, New York University, China; ^3^Key Laboratory of Neuroregeneration of Jiangsu and Ministry of Education, Co-Innovation Center of Neuroregeneration, Nantong University, China; ^4^Nantong Fourth People's Hospital, China

## Abstract

Hepatocellular carcinoma (HCC) is one of the most important causes of cancer-related deaths and remains a major public health challenge worldwide. Considering the extensive heterogeneity of HCC, more accurate prognostic models are imperative. The circadian genes regulate the daily oscillations of key biological processes, such as nutrient metabolism in the liver. Circadian rhythm disruption has recently been recognized as an independent risk factor for cancer. In this study, The Cancer Genome Atlas (TCGA) and Genotype-Tissue Expression (GTEx) were compared and 248 differentially expressed genes (DEGs) of the circadian rhythm were identified. HCC was classified into two subtypes based on these DEGs. The prognostic value of each circadian rhythm-associated gene (CRG) for survival was assessed by constructing a multigene signature from TCGA cohort. A 6-gene signature was created by applying the least absolute shrinkage and selection operator (LASSO) Cox regression method, and all patients in TCGA cohort were divided into high- and low-risk groups according to their risk scores. The survival rate of patients with HCC in the low-risk group was significantly higher than that in the high-risk group (*p* < 0.001). The patients with HCC in the Gene Expression Omnibus (GEO) cohort were also divided into two risk subgroups using the risk score of TCGA cohort, and the overall survival time (OS) was prolonged in the low-risk group (*p* = 0.012). Based on the clinical characteristics, the risk score was an independent predictor of OS in the patients with HCC. The Gene Ontology (GO) and Kyoto Encyclopedia of Genes and Genomes (KEGG) analyses showed that multiple metabolic pathways, cell cycle, etc., were enhanced in the high-risk group. Using the metabolic pathway single-sample gene set enrichment analysis (ssGSEA), it was found that the metabolic pathways in the high- and low-risk groups between TCGA and GEO cohorts were altered essentially in the same way. In conclusion, the circadian genes play an important role in HCC metabolic rearrangements and can be further used to predict the prognosis the patients with HCC.

## 1. Introduction

Liver cancer is the sixth most commonly diagnosed cancer and the fourth leading cause of cancer-related deaths worldwide. Hepatocellular carcinoma (HCC) is the most common type of liver cancer because it is clinically and biologically heterogeneous and hence unresponsive to most conventional treatments; the 5-year overall survival rate remains below 20% [1]. Hepatectomy is an effective method to eradicate early-stage HCC. Additionally, transarterial chemoembolization (TACE) is recommended for patients with unresectable HCC, but it is not yet satisfactory in improving the patient's prognosis [2]. As HCC is a highly heterogeneous cancer, the conventional models that use clinical tumor lymph node metastases (TNMs), vascular infiltration, and other factors to predict mortality are still not able to provide satisfactory predictions. Therefore, it is imperative to develop effective prognostic models.

The circadian rhythm is an intrinsic, 24-hour time-keeping system that operates in all the cells of the body and governs the rhythmicity of the cellular functions, including metabolism, gene expression, and trafficking and transport of the cellular proteins. The hypothalamic suprachiasmatic nucleus (SCN) acts as the central clock that receives time signals from the light and synchronizes the peripheral clock through the neural, somatic, and behavioral pathways [3–6]. In the mammalian cells, SCN regulates the circadian rhythm through the core interlocked transcriptional-translational feedback loops (TTFLs) and clock-controlled genes enabling the tissues to adapt to their biological functions and predict the external changes. The core clock consists of transcriptional activators, brain and muscle ARNT-like 1 (BMAL1) and circadian locomotor output cycles kaput (CLOCK) (or neuronal PAS domain protein 2 (NPAS2)), and transcriptional repressors, Period (Per1/2) and Cryptochrome (Cry1/2); the primary TTFL is stabilized by the clock-controlled genes [6, 7]. In recent years, the World Health Organization (WHO) has designated circadian disruption as a possible carcinogen based on both population and laboratory findings. The loss of circadian control is also associated with poor treatment efficacy and early mortality among patients with cancer, such as cancers of the breast, colon, liver, prostate, pancreas, ovary, and lung [8–12]. Circadian gene dysregulation affects key cancer development and the progression pathways, including metabolic regulation, cell cycle control, apoptosis, and DNA damage response [13].

The peripheral tissue clock is regulated by SCN and the hepatocyte clock, and the hepatocyte clock appears to occupy a higher position in the peripheral clock hierarchy [14]. The liver is a central metabolic organ that governs the whole-body homeostasis, and the circadian rhythm plays a major role in liver homeostasis, including hepatic metabolism. Over 50% of liver metabolites have a circadian rhythm that is coupled with transcription of the clock genes [15]. Hepatocyte clock-metabolism crosstalk has been found to play an important role in the liver. For example, the core circadian molecule NPAS2 plays an important role in reprogramming glucose metabolism in HCC cells through upregulation of the glycolytic genes and downregulation of peroxisome proliferator-activated receptor-*γ* coactivator 1-*α* (PGC-1*α*) expression; BMAL1 in the mouse liver increases fatty acid oxidation and partially reduces ethanol-induced fatty liver and liver injury through overexpression of the adipogenic transcription factor carbohydrate-response element-binding protein (ChREBP) [16–18].

In this study, the transcriptome profiles and clinical information of patients with HCC were obtained from The Cancer Genome Atlas (TCGA) and Genotype-Tissue Expression (GTEx) and the circadian rhythm-related genes (CRGs) from the Explore the Molecular Signatures Database (MSigDB). After Cox regression analysis of the differential CRGs, the CRGs associated with overall survival (OS) were identified and a risk model was developed. The risk model was used to determine the metabolism-related features of HCC to better understand the interactions between the circadian rhythm and metabolic rearrangement in HCC as well as the mechanism of the progression of malignant HCC. Therefore, the risk model will further be able to guide the prognosis and treatment of HCC.

## 2. Materials and Methods

### 2.1. Datasets

The RNA sequencing (RNA-seq) data of 371 patients with HCC with clinical information and 50 normal samples were obtained from TCGA database (https://portal.gdc.cancer.gov/repository) on 25 June 2021. To increase the number of normal liver samples, we obtained RNA-seq data for 110 normal liver samples in the GTEx dataset at UCSC Xena (https://xenabrowser.net/datapages/). We use a perl script for data processing to logarithmically scale the FPKM values to log 2 (1 + FPKM). GSE14520 is a dataset of mRNA expression in tumors and paired nontumor tissues from patients with liver cancer. The data from the comprehensive Gene Expression Omnibus (GEO) (https://www.ncbi.nlm.nih.gov/) was downloaded from the GPL3921 platform including 216 tumor and 220 nontumor samples and their clinical information. The baseline characteristics of patients with HCC in TCGA and cohort GSE14520 are shown in Table S1.

### 2.2. Identification of Differentially Expressed Circadian Rhythm-Related Genes

248 genes related to the circadian rhythm were extracted from 13-gene sets in MSigDB, and they are presented in Table S2-3. To obtain more accurate DEGs between the normal and tumor tissues, 110 normal liver samples were obtained from the GTEx portal to expand the number of normal samples. The expression data were normalized to fragment per kilobase million (FPKM). Differential expression analysis was performed using the Wilcoxon test, and differential CRGs were identified with *p* < 0.01 and |log2FC| ≥ 1.

### 2.3. Construction of the Protein-Protein Interaction (PPI) Network

The PPI network was constructed using the STRING database (https://string-db.org/) and visualized using the Cytoscape 3.8.2 software, and the hub genes were identified using Cytoscape Apps CytoHubba. To build the correlation network of the CRGs, the “igraph” package in R was used.

### 2.4. Consensus Clustering of Circadian Rhythm-Related Genes

A consistent cluster analysis was performed on the 54 DEGs listed in Table S4 using the “ConsensusClusterPlus” package. The number of clusters is denoted by the letter “k” from *k* = 2 to 9. It was determined that *k* = 2 had the best clustering stability. Finally, the patients with HCC were classified into two subtypes.

### 2.5. Development and Validation of the CRG Prognostic Model

To assess the prognostic value of DEGs, Cox regression analysis was used to assess the correlation between each gene and survival status in TCGA cohort. The cut-off of *p* value was 0.01, and the 10 survival-related genes were selected for further analysis. In order to narrow down the candidate genes and develop the prognostic model, LASSO Cox regression analysis (R package “glmnet”) was used. In the end, 6 genes and their coefficients were retained, and the penalty parameter (*λ*) was decided according to the minimum criteria. After centralization and standardization (using the “scale” function in R) of TCGA expression data, the risk score formula used was as follows: *risk* *score* = *Σ*6*i* *Xi* × *Yi* (*X* is coefficients, and *Y* is gene expression level). According to the median risk score, the patients with HCC in TCGA cohort were divided into low- and high-risk subgroups, and the OS time was compared between the two subgroups via Kaplan–Meier analysis. The “prcomp” function provided by the “Rtsne” package in the R was used to calculate the PCA and t-SNE based on the 6-gene signature. For the analysis of ROC curves, the “survival,” “survminer,” and “time-ROC” R packages were used. An HCC cohort from GEO (GSE14520) was implemented in the validation studies. Similarly, the expression levels of DEGs were normalized using the “scale” function, and the risk scores were calculated the same way as TCGA cohort. The GSE14520 cohort risk score was calculated based on the risk score formula for TCGA cohort, and categorized patients were divided into low- and high-risk subgroups to validate the genetic model.

### 2.6. Independent Prognostic Assessment of the Risk Score

The patients' clinical information on TCGA and GEO cohorts was extracted (age, grade, stage, gender, AFP, etc.). In our regression model, these variables were analyzed in conjunction with the risk score. The analysis was conducted using both univariate and multivariate Cox regression models.

### 2.7. Functional Enrichment Analysis of the DEGs between the Subgroups

TCGA cohort of patients with HCC was stratified into two subgroups according to the median risk score. A comparison of DEGs between the low- and high-risk groups was filtered based on a set of specific criteria (|log2FC| ≥ 1 and FDR < 0.05). The GO and KEGG analyses based on these DEGs were performed using the OmicShare tool, a free online data analysis platform (https://www.omicshare.com/tools). The ssGSEA was conducted with the “gsva” package to calculate the assessment of metabolism-associated pathway expression levels [19].

### 2.8. Statistical Analysis

One-way analysis of variance (ANOVA) was used to compare the gene expression levels between the normal liver and HCC cancer tissues, while the Pearson chi-square test was used to compare the categorical variables. Based on a two-sided log-rank test, Kaplan–Meier method was employed to compare OS between the patient subgroups. The univariate and multivariate Cox regression models were used to assess the independent prognostic value of the risk model. Comparing metabolism-associated pathway expression levels between the two groups was carried out by using the Mann–Whitney test. We conducted all statistical analyses with the R software (v4.0.2).

## 3. Results

### 3.1. Comparative Analysis of the CRGs from the Normal and Cancerous Tissues

**Figure 1:**
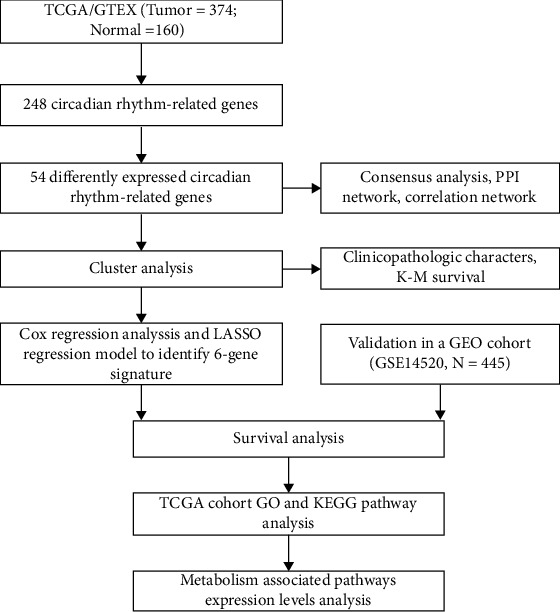
The flow diagram of the integral analysis. Gene expression profiles and corresponding clinical data were obtained from TCGA and GTEx for tumor and normal tissues, excluding samples with incomplete information. Differential expression analysis was performed to identify 53 differentially expressed circadian rhythm-associated genes. Next, HCC patients in TCGA dataset were classified into two distinct molecular subtypes (C1 and C2 clusters) by consistent clustering analysis of these 53 genes. The two HCC molecular subtypes differed significantly in terms of clinicopathological characteristics and overall survival. We then used Cox regression analysis and LASSO regression to build a risk model based on six circadian rhythm-related genes, and we used TCGA cohort and the GEO cohort as the training and validation sets for the model, respectively. Finally, variance analysis, enrichment analysis, and ssGSEA were performed based on the risk model.

**Figure 2:**
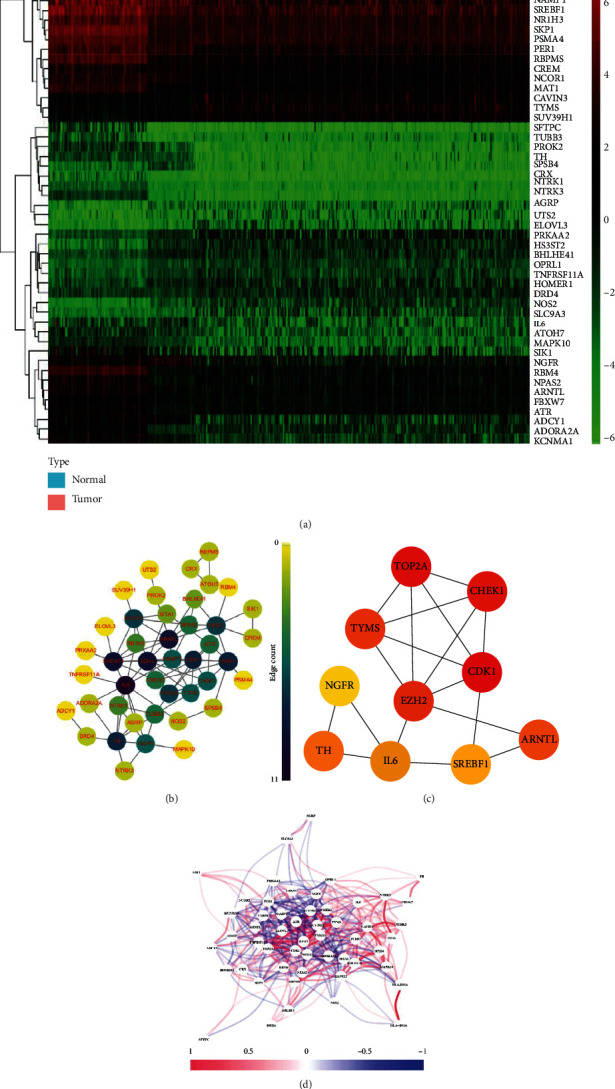
Expression of the circadian rhythm-related genes (CRGs) and their interactions. (a) Heatmap (green: low expression level; red: high expression level) of the CRGs between the normal (N, brilliant blue) and the tumor tissues (T, red). (b) Protein-protein interaction (PPI) network showing the interactions of the CRGs. (c) Calculation of the hub genes in PPI based on CytoHubba plug-in. (d) The correlation network of the CRGs (red line: positive correlation; blue line: negative correlation. The depth of the colors reflects the strength of the relevance).

The flow diagram of the integral analysis is shown in Figure 1. Out of the obtained 248 CRGs expressed in 371 patients with HCC and 160 controls, 54 DEGs (*p* < 0.01) were identified. Additionally, 23 genes (HOMER1, EZH2, CHEK1, TYMS, NOS2, CDK1, TOP2A, SLC9A3, etc.) were overexpressed in the tumor group while 31 genes (PROK2, ADORA2A, NPAS2, SREBF1, ATOH7, PER1, NTRK1, HOMER1, etc.) were downregulated. A heatmap of the RNA levels for these genes is shown in Figure 2(a). For a deeper understanding of the interactions between these CRGs, the protein-protein interaction (PPI) network was analyzed (Figure 2(b)). It was determined that TOP2A, TYMS, NGFR, TH, CHEK1, EZH2, IL6, CDK1, SREBF1, and ARNTL were the hub genes (Figure 2(c)). The correlation network containing these DEGs is shown in Figure 2(d).

### 3.2. Classification of the Tumors Based on the DEGs

**Figure 3:**
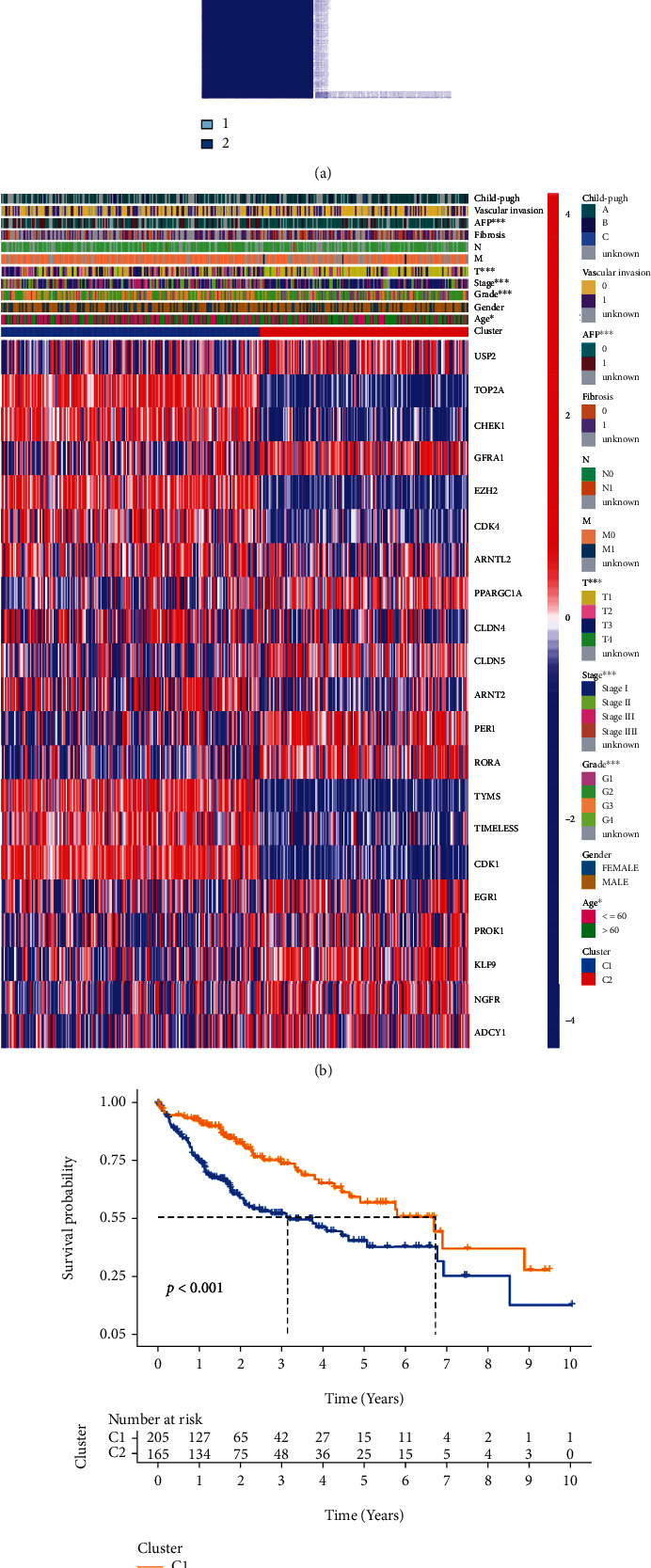
Tumor classification based on the pyroptosis-related DEGs. (a) 371 patients with HCC were grouped into two clusters according to the consensus clustering matrix (*k* = 2). (b) Heatmap and the clinicopathologic characters of the two clusters classified by these DEGs (^∗^*p* < 0.05; ^∗∗∗^*p* < 0.001). (c) Kaplan–Meier OS curves for the two clusters.

Consensus clustering analysis was performed on all 371 patients with HCC from TCGA cohort to investigate the association between the expression of DEGs and HCC subtypes. With the clustering variable (*k*) increased from 2 to 9, the intragroup correlations increased while the intergroup correlations dropped, indicating that the patients with HCC could be well divided into two clusters based on 54 DEGs (Figure 3(a)). In the heatmap, gene expression profiles and clinical characteristics such as age (≤60 or >60 years), AFP (≤300 or >300 ng/mL), stage (I–IV), and grade (G1–G4) had significant differences (Figure 3(b)). There was a significant difference in OS between the two clusters (*p* < 0.001, Figure 3(c)).

### 3.3. Modeling of Prognostic Gene Expression in TCGA Cohort

**Figure 4:**
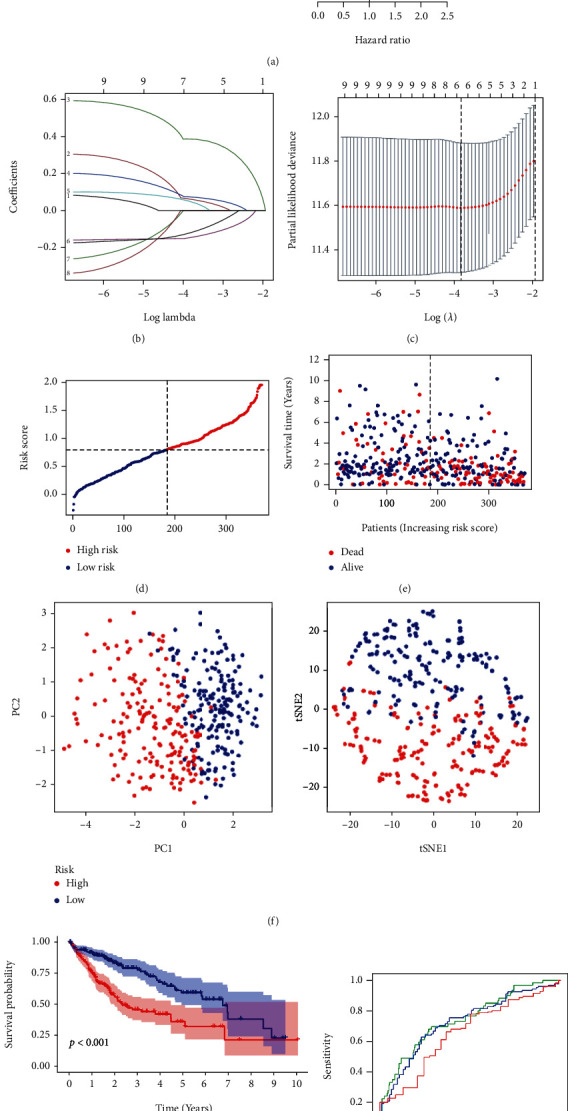
Constructing risk characteristics in TCGA cohort. (a) Univariate Cox regression analysis of OS for each DESs with *p* < 0.01 for 10 genes. (b) LASSO regression of 10 OS-associated genes. (c) Cross-validation adjusted parameter selection for LASSO regression. (d) Patient distribution based on the risk score. (e) Survival status of each patient (low-risk population: left side of the dashed line; high-risk population: right side of the dashed line). (f) PCA and t-SNE plot for HCC based on the risk score. (g) Kaplan–Meier curve groups for OS in the high- and low-risk patients. (h) The ROC curves show the predictive efficiency of the risk score.

In total, 371 HCC samples were matched with the corresponding patients whose survival records were available. For the primary screening of the survival-related genes, Cox regression analysis was used. 10 genes (TOP2A, CHEK1, EZH2, CDK4, ARNTL2, PPARGC1A, PER1, TYMS, TIMELESS, and CDK1) met the *p* < 0.01 criteria and were, thus, retained for further analysis. Among them, 8 genes (TOP2A, CHEK1, EZH2, CDK4, ARNTL2, TYMS, TIMELESS, and CDK1) were associated with increased risk with hazard ratios (HRs) > 1; the other two genes (PPARGC1A and PER1) were protective with HRs < 1 (Figure 4(a)). Based on the optimum *λ* value, a 10-gene signature was constructed by using the least absolute shrinkage and selection operator (LASSO) Cox regression analysis (Figures 4(b) and 4(c)). The risk score was calculated as follows: risk score = (0.058∗CHEK1 exp.) + (0.38∗EZH2 exp.) + (0.074∗CDK4 exp.) + (0.046∗ARNTL2 exp.) + (−0.14∗PPARGC1A exp.) + (−0.11∗PER1 exp.). The risk score formula calculated the median score of 371 patients, which was divided evenly into low- and high-risk subgroups (Figure 4(d)). In the high-risk group, more deaths and a shorter survival time were observed than in the low-risk group (Figure 4(e)). Based on the principal component analysis (PCA) and t-distributed Stochastic Neighbor Embedding (tSNE), patients with different risk scores were divided into two clusters (Figure 4(f)). Also, the OS time differed significantly between the two groups (*p* < 0.001, Figure 4(g)). To evaluate the sensitivity and specificity of the prognostic model, the time-dependent receiver operating characteristic (ROC) analysis was used, and it shows that the area under the curve (AUC) was 0.741 for 1-year, 0.724 for 3-year, and 0.655 for 5-year survival (Figure 4(h)).

### 3.4. External Validation of the Risk Signature

**Figure 5:**
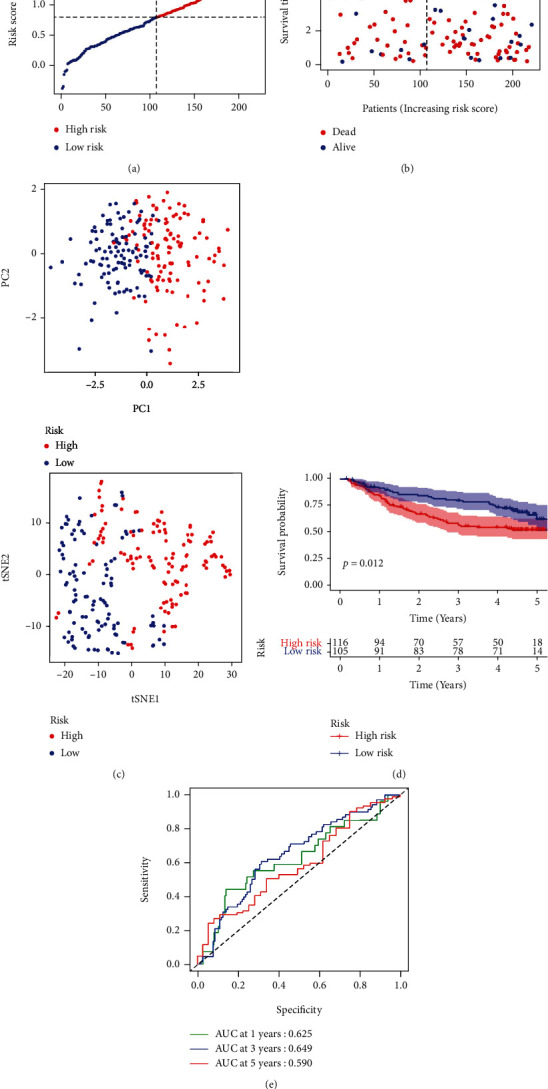
Validation of the risk model in the GEO cohort. (a) Distribution of the patients in the GEO cohort based on the median risk scores in TCGA. (b) Survival status of each patient (low-risk population: left side of the dashed line; high-risk population: right side of the dashed line). (c) PCA and t-SNE plot of HCCs. (d) Kaplan–Meier curves were used to compare OS in the low- and high-risk groups. (e) Time-dependent ROC curves for HCCs.

The normalized gene expression data from the GEO cohort (GSE14520) of 216 patients with HCC was used as a validation cohort. According to the median risk score in TCGA cohort, 105 patients in the GEO cohort were classified as low-risk, while the other 116 patients were classified as high-risk (Figure 5(a)). The results showed that low-risk patients (Figure 5(b), to the left of the dashed line) lived longer and had lower mortality than those in the high-risk group. The separation of the two subgroups was satisfactory by the PCA and t-SNE (Figure 5(c)). The Kaplan–Meier analysis indicated a significant difference in the survival rates between the two groups (*p* = 0.012, Figure 5(d)). In the ROC curve analysis of the GEO cohort, it was found that this model was a good predictor of event outcome (AUC = 0.625 for 1-year, 0.645 for 3-year, and 0.59 for 5-year survival) (Figure 5(e)).

### 3.5. Independent Prognostic Value of the Risk Model

**Figure 6:**
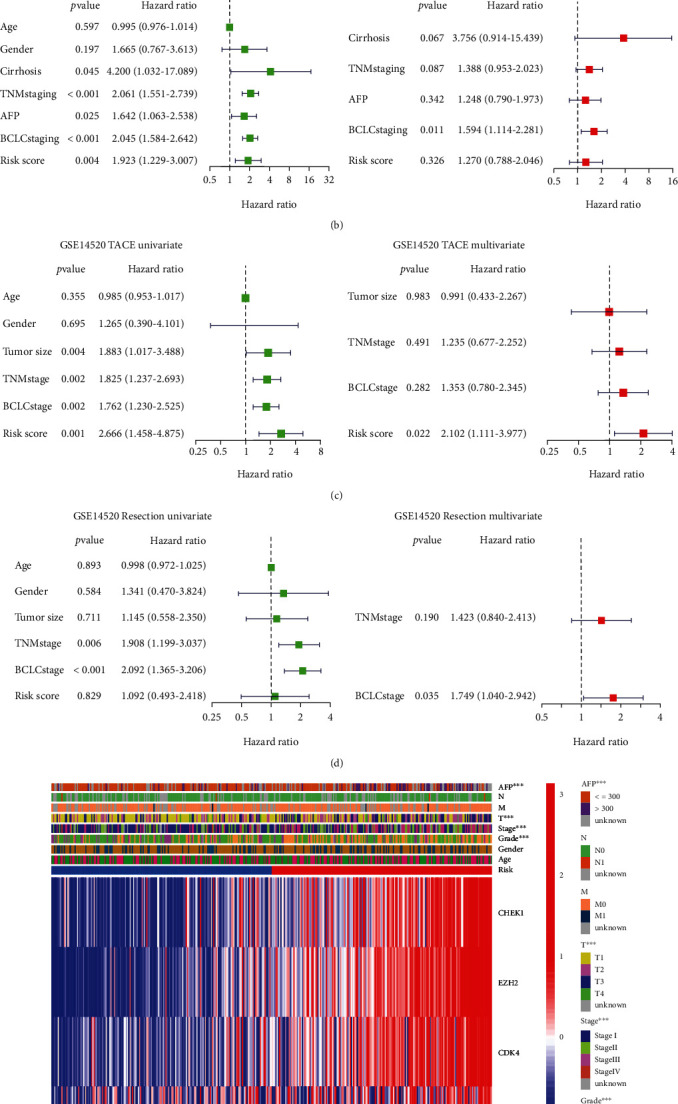
Univariate and multivariate Cox regression analyses of the risk scores. (a) Univariate and multivariate analyses for TCGA cohort. (b) Univariate and multivariate analyses for the GEO cohort. (c) Univariate and multivariate analyses for the GSE14520 TACE group. (d) Univariate and multivariate analyses for the GSE14520 resection group. (e) Heatmap (green: low expression; red: high expression) for the connections between clinicopathologic features and the risk groups (^∗∗∗^*p* < 0.001).

The univariate and multivariate Cox regression analyses were used to assess whether the risk score derived from the 10-gene signature model could be used as an independent prognostic factor. The univariate Cox regression analysis indicated that the risk score was an independent factor predicting poor survival in both TCGA and GEO cohorts (HR = 2.667, 95% confidence interval (CI): 1.548–4.596 and HR: 1.950, 95%, CI: 1.237–3.072, respectively, Figures 6(a) and 6(b)). Furthermore, multivariate analysis of the results showed that the risk score was a significant prognostic factor for patients with HCC in TCGA cohort but not in the GEO cohort after controlling for other confounding factors (HR = 2.043, 95% CI: 1.115–3.743, Figures 6(a) and 6(b)). As circadian dysregulation is strongly associated with the efficacy of cancer chemotherapy [12, 20], the GSE14520 dataset was divided into transcatheter arterial chemoembolization (TACE) and surgical resection groups according to the treatment modalities [21]. The univariate and multivariate Cox regression analyses were used to assess whether the risk scores from the genetic signature models can be used as independent prognostic factors for the patients with HCC under different treatment modalities. The analyses showed that the risk score was an independent predictor of poor prognosis in the TACE treatment group, whereas in the surgery group, it was not a prognostic factor (TACE group univariate HR: 2.666, 95% CI: 1.458–4.875 and multivariate HR: 2.102, 95% CI: 1.111–3.977, Figures 6(c) and 6(d)). In addition, a heatmap of the clinical characteristics of TCGA cohort (Figure 6(e)) was generated and differences between the low- and high-risk subgroups were found in terms of patient age, alpha-fetoprotein (AFP), stage, and grade.

### 3.6. Risk Model-Based Functional Analyses

**Figure 7:**
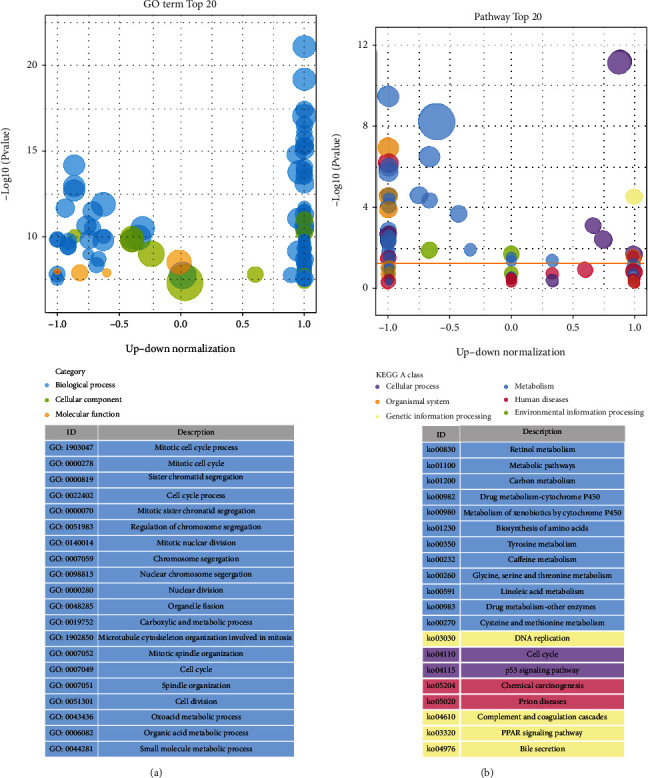
Functional analysis based on the DEGs between the two risk groups in TCGA cohort. (a) Bubble graph for GO enrichment (the bigger bubble means the more genes enriched). (b) Bubble graph for KEGG enrichment (the bigger bubble means the more genes enriched, and the color of the bubble represents the class type of the pathway).

Further, the differences in the gene pathways and functions between subgroups were explored using the “limma” R-package in order to extract DEGs by applying the false discovery rate (FDR) < 0.05 and |log2FC| ≥ 1 criteria. A total of 184 DEGs were identified between the low- and high-risk groups in TCGA cohort. Within the high-risk group, 77 of these genes were upregulated, while 107 were downregulated (Table S5). These DEGs were used to perform the Gene Ontology (GO) enrichment and Kyoto Encyclopedia of Genes and Genomes (KEGG) pathway analyses. The GO enrichment analysis showed that DEGs were mainly associated with the cell cycle and cell mitotic processes, and the results of KEGG analysis focused on multiple cellular metabolism-related and cell cycle-related signaling pathways (Figures 7(a) and 7(b)).

### 3.7. Analysis of the Metabolism-Associated Pathway Expression Levels between the Subgroups

**Figure 8:**
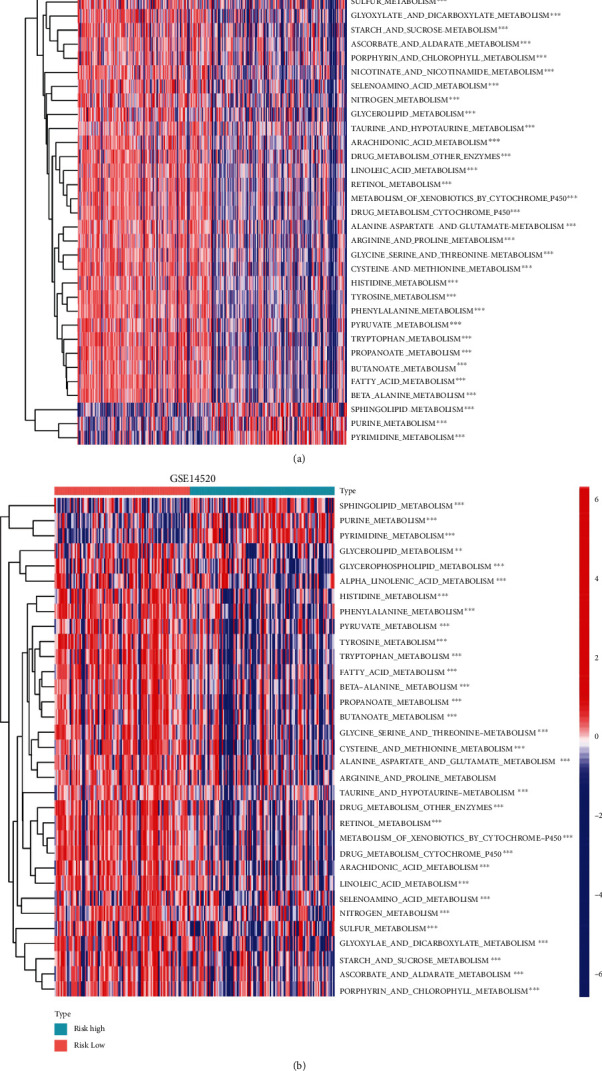
Comparison of the expression levels of metabolism-related pathways ssGSEA. Heatmap of the expression levels of 41 metabolism-related pathways in the low- and high-risk groups of TCGA and GEO cohort (^∗^*p* < 0.05; ^∗∗^*p* < 0.01; ^∗∗∗^*p* < 0.001).

After the functional analyses on TCGA cohort, the single-sample gene set enrichment analysis (ssGSEA) was done to analyze the metabolism-associated pathway expression levels between the low- and high-risk groups, with reference to the method of Zhang et al. [19]. TCGA cohort showed that 34 metabolic pathway expression levels differed between the two groups, with 31 metabolic pathway expression levels downregulated and 3 upregulated in the high-risk group. A total of 33 metabolic pathways were altered in the GEO cohort, and those altered in the high-risk and low-risk groups were consistent with TCGA cohort, apart from “niacin and nicotinamide metabolism,” which were downregulated in TCGA cohort. There was a striking similarity in the changes in the metabolic patterns in different HCC datasets after grouping according to this risk model (Figure 8).

## 4. Discussion

The physiological activities of the human body are affected by the circadian rhythm. However, this process is interrupted in tumors, such as breast and prostate cancer. Because of circadian disruption, the constitutive aldosterone receptor (CAR) is activated, leading to cholestasis and tumor in the liver. This overexpression of CAR ultimately leads to the progression from nonalcoholic fatty liver disease (NAFLD) to nonalcoholic steatohepatitis (NASH) and, eventually, to HCC [16]. In this study, a prognostic model was constructed, and it was demonstrated that abnormal circadian rhythm is associated with altered metabolic pathways of HCC. In the study, the expression levels of 248 genes known to be related to the circadian rhythm were examined first in both normal and HCC tissues, and it was found that 54 genes were differentially expressed significantly. Accordingly, based on the consensus clustering analysis of the DEGs, the clinical characteristics of the two clusters also showed significant differences. In order to assess the prognostic value of these CRGs further, an additional risk score model with a 6-gene risk signature was constructed using Cox univariate analysis and LASSO Cox regression analysis, which was then validated to perform well in an external dataset. The functional analyses indicated that the DEGs between the low- and high-risk groups had altered metabolic characteristics. In TCGA and GEO datasets, 33 metabolic pathways with altered expression had the same pattern of expression in high- and low-risk subgroups.

Epidemiological studies have shown that circadian rhythm disturbances (e.g., jet lag, shift work, sleep disruption, and exposure to the light at night) are associated with increased risk of cancer, including prostate, breast, colon, liver, pancreatic, ovarian, and lung cancers [22–25]. The relationship between the dysregulation of the circadian genes and the development and malignant progression of HCC has been confirmed by studies in animal models. The knockdown of PER2 increases c-Myc expression and disrupts clock-controlled pathways and patterns; complete knockdowns of Cry1 and Cry2 also disrupt the molecular circadian clock and increase the chemically induced hepatocarcinogenesis. The analysis of the miRNA profiles in the liver of the Clock mutant mice shows that Clock-regulated miRNAs may be involved in cancer development or progression by controlling genes that are involved in cell proliferation, invasion, and/or metabolism. These studies suggest that the normal circadian rhythm has the potential to suppress tumors while disrupted circadian rhythm is an important risk factor for HCC. Therefore, the intervention of the circadian rhythm may be an important strategy to prevent the development of HCC and to design novel treatment options. In HCC, the interactions of CRGs and whether they are related to the survival time of the patients remain unknown. Our study discovered that a signature featuring six CRGs (CHEK1, EZH2, CDK4, ARNTL2, PPARGC1A, and PER1) could be used to predict the OS of the patients with HCC. CHEK1, a member of the CHEK family, is a serine/threonine-specific protein kinase; it is mainly involved in the coordination of DNA repair and is, therefore, an area of great interest in cancer development and therapy [26]. This gene can affect the downstream signaling pathways of TP53 and CHEKs through activation of ataxia-telangiectasia mutation (ATM)/ataxia-telangiectasia and Rad3-related protein (ATR), leading to G2/M blockade and initiation of DNA repai [27, 28]. This gene has been shown to be overexpressed in several solid tumors. The correlation of CHEK1 expression with the tumor grade and disease recurrence has also been reported, suggesting a role in tumor development, which may be a result of its regulatory role in the circadian rhythm [29]. EZH2 (enhancer of zeste homolog 2) is a member of the Polycomb histone gene (PcGs) family, which is a group of important epigenetic regulators that induce chromatin-mediated transcriptional repression through deacetylation and methylation of histone H3. EZH2 regulates cell cycle progression, and the dysregulation of EZH2 accelerates cell proliferation and prolonged cell survival, which may lead to cancer development [30]. It was shown that EZH2 is a component of the CLOCK-BMAL1 complex, and that EZH2 enhances CRY protein-mediated transcriptional repression in HCC [31]. The high expression of EZH2 is associated with poor survival outcomes. The cell cycle protein-dependent kinases (CDKs) were discovered first in 1970, and 40 years later, the first small molecule CDK inhibitors were approved for the treatment of human cancers. A recent genome-scale clustered regularly interspaced short palindromic repeats (CRISPR)-dCas9 activation screen in bladder cancer cells showed that CDK4/6 inhibitors bind effectively to a variety of compounds targeting PI3K, AKT, mTOR, FGFR1/2, MEK1/2, VEGFR1/2/3, PDGFRb, cKIT, and even pan-CDK inhibitors [32]. It has shown that long-term circadian rhythm disruption promotes proproliferative signaling events that stimulate G1/S phase progression through CDK4/6-dependent aggregation of retinoblastoma protein phosphorylation [33]. In this study, CDK4 showed procancer effects as it was upregulated in the HCC tissues, and then, it further shortened the lifespan of the patients with HCC as it was enriched in the high-risk group. ARNTL2 encodes a basic helix-loop-helix transcription factor belonging to the PAS (PER, ARNT, and SIM) superfamily. PAS proteins play an important role in the adaptation to low atmospheric and cellular oxygen levels, exposure to certain environmental pollutants and circadian oscillations of the light and temperature. ARNTL2 is involved in tumor progression. It is associated with aggressive, metastatic, and invasive colorectal and breast cancers [34]. ARNTL2 induces complex primary metastatic secretions and makes metastasis self-sufficient in lung adenocarcinoma [35]. In this study, ARNTL2 was highly expressed in tumors and predicted a poor prognosis. PPARGC1A encodes PGC-1*α* and is a multifunctional transcriptional coactivator; it is involved in many metabolic processes and has been associated with several human diseases, e.g., it activates the transcription factor peroxisome proliferator-activated receptor-*α* (PPAR*α*) to enhance fatty acid metabolism [36]. In addition, PPARGC1A binds the transcription factors hepatocyte nuclear factor-4*α* (HNF-4*α*) and glucocorticoid receptor to stimulate gluconeogenesis [37]. It was found that PPARGC1A is downregulated in the tumor tissues, and its low expression predicts poor survival, suggesting its function as a tumor suppressor gene. The situation of PPARGC1A and PER1 is similar in this study. PER1 is a member of the Period family of genes and is expressed in a circadian pattern in the SCN, the primary circadian pacemaker in the mammalian brain. PER1 is upregulated by CLOCK/ARNTL heterodimers but then it represses this upregulation in a feedback loop using PER/CRY heterodimers to interact with CLOCK/ARNTL. Polymorphisms in this gene may increase the risk of developing certain cancers. PER1 is the most common clock gene that is implicated in the endocrine neoplasms; in most cases, their expression is downregulated in tumoral compared to normal tissues, including in HCC [38]. In general, 6 genes in the prognostic model were identified as possible circadian rhythm executors or promoters in patients with HCC. However, these promoters and executors were not all associated with better HCC prognosis in this study. How these genes interact with each other during dysregulation of the circadian rhythm in HCC remains to be further investigated.

Altered metabolism is a well-recognized hallmark of cancer [39]. The adverse effects of circadian dysregulation on hepatic metabolism have been extensively studied. In fact, more than 50% of liver metabolites have a circadian rhythm, which is associated with clock gene transcription, and in the results of the two cohorts, both nucleotide and sphingolipid metabolism were upregulated in the high-risk group [15]. Nucleotide metabolism is typically increased in HCC and predicts the rapid proliferation of tumor cells. The biosynthesis of purine and pyrimidine requires the participation of glutamine, aspartic acid, glycine, and phosphoribosyl pyrophosphate. Therefore, inhibition of the urea cycle (one of the significant pathways of glutamine use) is also common in HCC because the promoter region of the urea cycle rate-limiting enzyme, carbamylphosphatase synthetase-I, is highly methylated in HCC, leading to reduced levels of mRNA expression in the cell lines and tumors, resulting in increased nucleotide synthesis because of the elevated glutamine levels [40]. In many studies, sphingolipids including two central bioactive lipids, ceramide and sphingosine-1-phosphate (S1P), have been shown to cause apoptosis at elevated ceramide levels and to inhibit apoptosis at elevated S1P levels. The high expression of S1P has also been associated with resistance to various tumor drugs, a phenomenon observed in the TACE treatment group that may be related to sphingolipid metabolism [41, 42].

## 5. Conclusions

This study demonstrated that the circadian rhythm is closely associated with HCC, as most of the CRGs between the normal and HCC tissues were differently expressed. Moreover, the score generated from the novel risk signature based on the six CRGs was an independent risk factor for predicting OS in both TCGA and GEO cohorts. The DEGs between the low- and high-risk groups were associated with metabolism-associated pathway expression levels. Our study provides a novel gene signature for predicting the regulation role of the circadian rhythm in patients with HCC and offers a significant basis for future studies of the relationships between CRGs and metabolism in HCC.

## Data Availability

All data was obtained from the public database described in Materials and Methods.

## Supplementary Materials

Supplementary Materials Supplementary Table 1: clinical features of hepatocellular carcinoma patients from TCGA database and GSE14520. Supplementary Table 2: circadian rhythm-associated gene set from MSigDB. Supplementary Table 3: circadian rhythm-related genes. Supplementary Table 4: differential circadian rhythm-related genes in cancer and normal. Supplementary Table 5: differential genes between the low- and high-risk groups.
