# Supplementary material for: Development and Validation of a Novel Circadian Rhythm-Related Signature to Predict the Prognosis of the Patients with Hepatocellular Carcinoma
Source: Biomed Res Int. 2022 Aug 12;2022:4263261. doi: 10.1155/2022/4263261 (PMC9391189; doi:10.1155/2022/4263261)
Supplement: Supplementary Materials — Supplementary Table 1: clinical features of hepatocellular carcinoma patients from TCGA database and GSE14520. Supplementary Table 2: circadian rhythm-associated gene set from MSigDB. Supplementary Table 3: circadian rhythm-related genes. Supplementary Table 4: differential circadian rhythm-related genes in cancer and normal. Supplementary Table 5: differential genes between the low- and high-risk groups. [file 4263261.f1.pdf]

Clinical characteristics of HCC patients	TCGA (total 371)	GSE14520 (total 216)
**Age**		
≤60	176	178
> 60	195	38
**Gender**		
Female	120	29
Male	251	187
**BCLC staging**		
A		145
B		22
C		29
Unknown		20
**TNM staging**		
I	174	90
II	85	77
III	84	49
IV	4	
Unknown	24	
**T**		
T1	184	
T2	92	
T3	79	
T4	13	
Unknown	3	
**N**		
N0	253	
N1	4	
Unknown	114	
**M**		
M0	269	
M1	3	
Unknown	99	
**Grade**		
G1	55	
G2	178	
G3	120	
G4	13	
Unknown	5	
**Cirrhosis**		
Yes		198
No		18

**Fibrosis**		
Yes	139	
No	76	
Unknown	156	
**AFP**		
≤300	218	118
>300	64	98
Unknown	89	
**Vascular invasion**		
Yes	209	
No	108	
Unknown	54	
**Child–Pugh**		
A	222	
B	21	
C	1	
Unknown	127	
**Status**		
Alive	239	132
Dead	132	84

Supplementary Table 1: Clinical features of Hepatocellular carcinoma patients from TCGA database and GSE14520.

Circadian rhythm-associated gene set from MSigDB

BIOCARTA_CIRCADIAN_PATHWAY	Circadian Rhythms
GOBP_CIRCADIAN_SLEEP_WAKE_CYCLE_NON_REM_SLEEP	All sleep stages in the circadian sleep/wake cycle other than REM sleep. These stages are characterized by a slowing of brain waves and other physiological functions.
GOBP_ENTRAINMENT_OF_CIRCADIAN_CLOCK	The synchronization of a circadian rhythm to environmental time cues such as light.
GOBP_NEGATIVE_REGULATION_OF_CIRCADIAN_RHYTHM	Any process that stops, prevents, or reduces the frequency, rate or extent of a circadian rhythm behavior.
GOBP_REGULATION_OF_CIRCADIAN_RHYTHM	Any process that modulates the frequency, rate or extent of a circadian rhythm. A circadian rhythm is a biological process in an organism that recurs with a regularity of approximately 24 hours.
GOBP_POSITIVE_REGULATION_OF_CIRCADIAN_RHYTHM	Any process that activates or increases the frequency, rate or extent of a circadian rhythm behavior. [GOC:go_curators]
GOBP_REGULATION_OF_CIRCADIAN_SLEEP_WAKE_CYCLE	Any process that modulates the frequency, rate or extent of the circadian sleep/wake cycle.
KEGG_CIRCADIAN_RHYTHM_MAMMAL	Circadian rhythm - mammal
PID_CIRCADIAN_PATHWAY	Circadian rhythm pathway
REACTOME_BMAL1_CLOCK_NPAS2_ACTIVATES_CIRCADIAN_GENE_EXPRESSION	BMAL1:CLOCK,NPAS2 activates circadian gene expression
REACTOME_CIRCADIAN_CLOCK	Circadian Clock
WP_CIRCADIAN_RHYTHM_RELATED_GENES	Circadian rhythm related genes
WP_EXERCISEINDUCED_CIRCADIAN_REGULATION	Exercise-induced Circadian Regulation

Supplementary Table 2: Circadian rhythm-associated gene set from MSigDB

Circadian rhythm-related genes

ARNTL2	PPP1CA	CSF2	NKX2-1	RORA	CRTC2	SERPINE1	CHRM1	IL6	PAX4
ATOH7	PPP1CB	CSNK1D	NLGN1	RORB	CRTC3	SIRT1	CIART	JUN	PRF1
BHLHE40	PPP1CC	CSNK1E	NMU	RORC	CUL1	SKP1	CLDN4	JUND	PRKG2
CRTC1	RBM4	DRD2	NOCT	SFPQ	DBP	SMARCD3	CRX	KCND2	PRMT5
CRY1	RBM4B	DRD3	NONO	SIAH2	ELOVL3	SREBF1	CST3	KCNH7	PROK1
CRY2	SIK1	DRD4	NPS	SIN3A	EP300	TBL1X	DDC	KCNMA1	PROK2
FBXL21P	SOX14	EZH2	NPY2R	SPSB4	F7	TBL1XR1	DDX5	KDM5A	PROKR2
FBXL12	TP53	FBXW11	NR1D1	SRRD	HELZ2	TGS1	DHX9	KLF9	PTEN
FBXL17	USP2	FBXW7	NR1D2	SUV39H1	HIF1A	UBA52	DRD1	KMT2A	RELB
FBXL22	ADA	GHRH	NR1H3	SUV39H2	KLF15	UBB	DYRK1A	LEP	SETX
FBXL3	ADCY1	GHRHR	OPN3	TARDBP	MED1	UBC	EGR1	LGR4	SFTPC
FBXL6	ADORA1	GHRL	OPN4	THRAP3	MEF2C	USP46	EGR3	MAGED1	SIX3
FBXL8	ADORA2A	GSK3B	PASD1	TIMELESS	MEF2D	AANAT	FAS	MC3R	SLC6A4
GNA11	ADRB1	HCRTR2	PIWIL2	TOP2A	NAMPT	ADIPOQ	GFPT1	METTL3	SLC9A3
GNAQ	ARNTL	HDAC3	PPARA	UBE3A	NCOA1	AGRP	HCRTR1	MTNR1A	STAR
ID2	ATG7	HNF4A	PPARG	USP7	NCOA2	AHCY	HDAC1	MTTP	TH
MTA1	BTRC	HNRNPD	PPARGC1A	USP9X	NCOA6	AHR	HDAC2	MYBBP1A	TNFRSF11A
NR2F6	CCAR2	KDM2A	PRKAA1	ZFHX3	NCOR1	ARNT	HEBP1	NAGLU	TOP1
OPN5	CDK1	KLF10	PRKAA2	ATF2	NFIL3	ARNT2	HNF1B	NGFR	TPH1
PDE6B	CHRNB2	MAGEL2	PRKCG	AVP	NPAS2	ATF4	HNRNPU	NMS	TPH2
PER1	CIPC	MAPK10	PRKDC	BHLHE41	NR3C1	ATF5	HOMER1	NOS2	TYMS
PER2	CLOCK	MAPK8	PROX1	CARM1	NRIP1	BTBD9	HS3ST2	NTRK1	UTS2
PER3	CREB1	MAPK9	PSPC1	CHD9	RAI1	CARTPT	HTR7	NTRK3	UTS2R
PHLPP1	CREM	MTNR1B	PTGDS	CPT1A	RPS27A	CAVIN3	ID3	OGT	
PML	CRH	MTOR	ROCK2	CREBBP	RXRA	CDK4	ID4	OPRL1	

Supplementary Table 3: circadian rhythm-related genes

Circadian rhythm-related genes differentially expressed genes

Gene	Normal-mean	Tumor-Mean	logFC	pValue	FDR
CREM	8.218070784	3.680449044	-1.158917959	7.05E-39	6.42E-38
ATOH7	0.534751895	0.197945933	-1.43376326	1.59E-21	4.46E-21
RBM4	11.87287617	1.357816839	-3.128308679	5.52E-25	1.86E-24
ARNTL	4.594027297	1.769389019	-1.376508154	6.89E-33	4.14E-32
NCOR1	8.798152258	3.243113616	-1.439821001	1.27E-65	3.12E-63
SKP1	40.31971891	11.49048253	-1.811046197	9.12E-24	2.84E-23
HS3ST2	0.26735743	0.814819169	1.607710149	3.80E-30	1.99E-29
TH	0.274280724	0.044136279	-2.635616326	1.21E-62	7.43E-61
TOP2A	0.560045206	6.402659677	3.515056138	3.00E-63	3.69E-61
CHEK1	0.465651001	1.286271717	1.46587445	1.36E-46	2.79E-45
SIK1	4.146311116	0.833726733	-2.314181872	3.92E-26	1.51E-25
EZH2	0.996694396	2.625944533	1.397613322	1.40E-42	1.73E-41
NAMPT	48.87334826	13.96545855	-1.807185001	3.61E-37	2.78E-36
UTS2	0.050920227	0.225822519	2.148878609	1.59E-10	2.71E-10
NOS2	0.097227997	0.455408416	2.227717252	9.56E-40	9.41E-39
OPRL1	0.146823064	0.335468503	1.192098704	2.49E-22	7.38E-22
ELOVL3	0.035987726	0.253239804	2.814927321	4.50E-15	9.97E-15
HLA-DMA	7.58297499	20.0081208	1.399749803	1.47E-17	3.55E-17
ADORA1	0.20516662	0.771645651	1.911142469	0.025379505	0.02825049
RBPMS	21.53230751	4.907062512	-2.133571379	2.51E-46	4.75E-45
AGRP	0.063154935	0.232542199	1.880525168	2.42E-07	3.46E-07
CAVIN3	2.411583704	4.938063147	1.0339644	1.24E-10	2.11E-10
ARNT2	0.204549518	0.642143554	1.650445718	0.041567534	0.044653333
ADORA2A	2.015854228	0.560597653	-1.846353707	3.98E-27	1.58E-26
HOMER1	0.338093032	0.724946552	1.100454349	5.14E-11	8.97E-11
PROK2	0.480664824	0.03021231	-3.991822828	2.83E-56	1.16E-54
TUBB3	0.210174971	0.092312358	-1.186995164	3.95E-06	5.40E-06
SREBF1	52.85638195	19.02809091	-1.473946852	1.94E-33	1.26E-32
SUV39H2	0.870675109	1.821345391	1.064798149	1.35E-50	3.01E-49
PER1	20.01295678	7.802629737	-1.358901986	2.88E-43	4.17E-42
MAPK10	0.454864647	0.175118554	-1.377105358	1.28E-44	2.26E-43
DRD4	0.360871638	1.045073904	1.534047304	1.95E-08	2.89E-08
CRX	0.080957906	0.009197925	-3.137791673	9.62E-25	3.15E-24
FBXW7	2.510819041	1.21818364	-1.043426421	1.08E-50	2.66E-49
TYMS	2.259355887	7.319215249	1.695777437	1.12E-42	1.53E-41
ATR	2.985066286	1.306585024	-1.191961959	9.26E-22	2.62E-21
BHLHE41	0.350846437	0.977471658	1.478215159	9.82E-07	1.37E-06
NTRK1	0.110069473	0.044958732	-1.291741147	7.57E-40	7.76E-39
CDK1	0.444780757	3.640814241	3.033094855	8.84E-63	7.25E-61
PSMA4	24.60123262	12.00736529	-1.034810979	2.99E-25	1.03E-24

NPAS2	4.709008035	1.578297618	-1.577053906	2.26E-21	6.19E-21
KCNMA1	2.926402389	0.473986907	-2.626209044	2.76E-52	7.55E-51
NGFR	5.641944114	2.40630436	-1.229373243	1.18E-35	8.32E-35
SLC9A3	0.082325075	1.155006292	3.810424978	3.66E-15	8.26E-15
PRKAA2	0.280607854	1.505183184	2.423311781	7.67E-28	3.20E-27
MTA1	10.18571544	3.718747482	-1.453658626	6.50E-26	2.42E-25
SUV39H1	1.751084589	4.304476259	1.297588933	1.10E-54	3.88E-53
SFTPC	0.381889654	0.024531968	-3.960420978	2.64E-12	4.85E-12
ADCY1	3.075460197	1.283983977	-1.260175105	6.98E-37	5.20E-36
NR1H3	24.82123226	11.38950144	-1.123870144	1.22E-35	8.34E-35
SPSB4	0.141646922	0.03354119	-2.078293472	4.28E-40	4.57E-39
NTRK3	0.236931855	0.020523494	-3.529123906	1.23E-60	6.03E-59
IL6	1.351032933	0.31562181	-2.097794035	1.39E-12	2.62E-12
TNFRSF11A	0.186638975	0.405257822	1.118589741	6.77E-10	1.10E-09

Supplementary Table 4: Differential Circadian rhythm-related genes in cancer and normal tissues.

Differential genes between low and high risk group

Gene	Low-mean	High-mean	logFC	pValue	FDR
TAT	7.17717634	4.358036727	-2.819139613	5.75E-31	1.81E-28
SDS	7.009410329	4.297202767	-2.712207562	9.11E-19	1.66E-17
PCK1	8.081370232	5.460036568	-2.621333664	2.05E-28	2.48E-26
HPD	8.996143868	6.433542361	-2.562601507	4.52E-19	8.88E-18
SLC10A1	7.070036565	4.677157321	-2.392879244	6.64E-21	1.87E-19
CYP2A6	7.07959406	4.708411032	-2.371183028	8.38E-14	6.01E-13
HSD11B1	7.438304691	5.412753899	-2.025550791	1.75E-12	1.02E-11
CYP3A4	6.783322336	4.773492115	-2.009830221	5.67E-11	2.61E-10
SLC22A1	6.149128368	4.157739089	-1.991389279	6.39E-13	3.99E-12
CPS1	8.374410058	6.45406246	-1.920347598	8.98E-15	7.65E-14
ADH1B	8.62981779	6.752033682	-1.877784108	9.34E-17	1.14E-15
GLYAT	5.165214663	3.318719692	-1.846494971	2.34E-18	3.99E-17
SLC27A5	6.61317213	4.837543726	-1.775628404	2.33E-22	8.77E-21
CFHR4	5.905355204	4.134992054	-1.77036315	2.18E-19	4.51E-18
F9	7.469813621	5.75259643	-1.717217191	1.08E-16	1.31E-15
GYS2	4.97927577	3.280627591	-1.698648179	2.71E-19	5.55E-18
CTH	5.583253946	3.89856168	-1.684692266	9.54E-23	3.98E-21
HPR	7.720942656	6.110385149	-1.610557506	1.45E-15	1.43E-14
AQP9	7.473397599	5.885703327	-1.587694272	1.59E-10	6.83E-10
C6	7.181351695	5.603004392	-1.578347303	6.04E-19	1.15E-17
CYP2C9	7.128355635	5.551785945	-1.57656969	5.17E-17	6.56E-16
ALDOB	10.37906632	8.806393754	-1.572672563	2.23E-16	2.54E-15
HSD17B6	8.450771082	6.909986377	-1.540784705	4.69E-19	9.14E-18
IGFBP1	9.368901851	7.845570694	-1.523331157	1.59E-14	1.30E-13
SERPINC1	10.90268529	9.392174359	-1.510510935	5.27E-21	1.52E-19
HRG	9.307254088	7.798043744	-1.509210344	5.05E-13	3.21E-12
GNMT	6.074728479	4.5769753	-1.497753179	3.51E-11	1.67E-10
BHMT	7.248220892	5.75275567	-1.495465222	5.45E-11	2.52E-10
CYP2C8	6.414332082	4.931903276	-1.482428805	9.43E-14	6.70E-13
INSIG1	8.015669474	6.558093494	-1.45757598	9.21E-22	3.11E-20
SAA1	8.262571765	6.812757864	-1.449813901	1.15E-05	2.45E-05
HP	8.988000806	7.54729086	-1.440709946	1.56E-17	2.16E-16
C8A	7.329626821	5.963828064	-1.365798757	8.85E-20	1.99E-18
AKR7A3	5.791679995	4.480073506	-1.311606488	4.66E-13	2.98E-12
SPP2	6.501325287	5.191605098	-1.309720189	8.90E-08	2.57E-07
HAO2	4.966996118	3.657433089	-1.30956303	3.51E-09	1.25E-08
OTC	6.699497645	5.401524534	-1.297973111	1.09E-11	5.65E-11
PPARGC1A	4.087892992	2.795680004	-1.292212988	5.66E-23	2.50E-21
MT1X	6.941387963	5.649443575	-1.291944388	1.46E-09	5.47E-09

SLC38A4	7.196792054	5.9182128	-1.278579254	3.05E-18	5.03E-17
AFM	6.410301312	5.132914658	-1.277386654	3.83E-10	1.56E-09
CFHR5	6.653991521	5.379207934	-1.274783587	2.60E-08	8.13E-08
HAO1	7.501144094	6.232430468	-1.268713625	1.03E-19	2.27E-18
HPX	10.050085	8.790955809	-1.259129196	1.22E-20	3.22E-19
ANGPTL4	6.24483693	4.987095539	-1.257741391	9.26E-15	7.86E-14
FBP1	7.374396683	6.11987199	-1.254524693	3.01E-16	3.35E-15
CYP1A2	3.940808965	2.687159924	-1.253649042	2.29E-09	8.35E-09
UGT1A4	6.394339792	5.140723011	-1.253616782	4.83E-09	1.69E-08
AGXT	8.154936549	6.902117697	-1.252818852	3.85E-15	3.50E-14
APOF	5.180776559	3.929391263	-1.251385296	1.44E-11	7.35E-11
ABCB4	5.64912183	4.400318603	-1.248803227	1.91E-14	1.54E-13
CYP4A11	6.633871856	5.385329337	-1.248542519	6.05E-14	4.45E-13
CDO1	7.692117189	6.445296664	-1.246820525	3.96E-17	5.09E-16
LEPR	5.114302054	3.869437388	-1.244864665	6.33E-14	4.64E-13
CYP4F2	6.130451813	4.886819463	-1.24363235	2.34E-14	1.86E-13
MASP2	5.727082971	4.484158536	-1.242924436	5.72E-16	6.00E-15
RDH16	5.958293797	4.717718015	-1.240575782	7.94E-11	3.58E-10
HGD	7.722528965	6.483464114	-1.239064851	2.22E-22	8.46E-21
PDK4	5.147430332	3.91761025	-1.229820083	1.33E-15	1.31E-14
SLC27A2	6.784498679	5.554997857	-1.229500822	1.68E-17	2.31E-16
BAAT	8.322852605	7.096461028	-1.226391578	4.45E-18	6.98E-17
APCS	9.992579429	8.771899999	-1.22067943	2.80E-09	1.01E-08
FABP4	3.843491935	2.631435281	-1.212056655	2.09E-15	2.01E-14
LECT2	6.522745505	5.312832047	-1.209913458	2.97E-07	7.91E-07
SLC25A15	5.87660225	4.667245719	-1.209356531	9.19E-19	1.67E-17
ANXA10	4.329202615	3.130229569	-1.198973046	1.12E-15	1.12E-14
AZGP1	9.086539397	7.896592406	-1.189946991	2.48E-18	4.19E-17
PON1	6.86082231	5.673584738	-1.187237572	2.56E-14	2.02E-13
NR1I3	5.17676949	3.995517102	-1.181252388	1.65E-12	9.63E-12
ABCA8	3.787726354	2.617779387	-1.169946967	7.80E-17	9.64E-16
SEC14L2	5.011464601	3.846434315	-1.165030286	4.97E-15	4.40E-14
CES2	7.740248068	6.586306196	-1.153941871	2.03E-13	1.38E-12
DCXR	8.859635924	7.709822116	-1.149813808	1.65E-14	1.35E-13
C9	5.95360108	4.804970295	-1.148630785	6.13E-05	0.000118672
CD14	8.091795644	6.94317393	-1.148621714	1.62E-17	2.25E-16
APOC3	12.11990244	10.97914392	-1.140758515	7.73E-14	5.59E-13
CYP2B6	4.62696774	3.503652331	-1.123315409	5.65E-11	2.61E-10
C4BPA	9.670784837	8.549176197	-1.12160864	2.15E-11	1.06E-10
C7	4.308528192	3.191658179	-1.116870013	6.87E-08	2.02E-07
SULT2A1	8.125510028	7.009040903	-1.116469125	5.95E-08	1.76E-07
UGT2B15	6.345290003	5.230219595	-1.115070408	3.11E-08	9.58E-08

F13B	6.441932047	5.335205146	-1.106726901	8.60E-14	6.16E-13
PLG	8.693866332	7.589934588	-1.103931744	3.73E-14	2.85E-13
GBA3	3.987325319	2.885253072	-1.102072247	4.88E-11	2.27E-10
OGDHL	4.998397658	3.896920769	-1.101476889	2.53E-13	1.69E-12
ARG1	8.447255872	7.354285715	-1.092970158	5.22E-12	2.83E-11
KCNJ8	5.032786355	3.951831265	-1.08095509	3.36E-17	4.39E-16
GLS2	3.276151792	2.201450226	-1.074701566	1.51E-10	6.53E-10
AOX1	7.533986359	6.464114094	-1.069872265	2.35E-09	8.54E-09
GPT	5.154037039	4.088063719	-1.06597332	2.07E-18	3.58E-17
ABCB11	3.518966137	2.463452888	-1.055513249	1.99E-14	1.60E-13
PFKFB1	4.082814036	3.028609318	-1.054204718	2.98E-14	2.31E-13
CCL16	6.183297695	5.131145509	-1.052152186	9.37E-07	2.33E-06
KNG1	10.42042785	9.369513466	-1.050914389	3.07E-13	2.02E-12
GADD45B	5.985792076	4.935134044	-1.050658032	2.70E-21	8.45E-20
ADH1A	8.239004434	7.189057573	-1.049946861	1.76E-08	5.60E-08
TTR	10.21537481	9.176002662	-1.039372144	9.33E-11	4.16E-10
SLCO1B1	6.53392709	5.499701388	-1.034225702	7.49E-11	3.39E-10
C8B	7.746879465	6.713374543	-1.033504922	2.80E-15	2.61E-14
TDO2	6.707609456	5.677320754	-1.030288702	3.47E-07	9.15E-07
IGFBP2	7.215826295	6.187186104	-1.02864019	1.26E-06	3.09E-06
TSC22D3	5.100871134	4.076703617	-1.024167517	2.71E-18	4.53E-17
GADD45G	5.292187471	4.269614909	-1.022572562	7.74E-17	9.60E-16
EHHADH	6.713459365	5.692493398	-1.020965968	2.23E-15	2.13E-14
AKR1D1	5.205414936	4.187338675	-1.018076261	1.11E-06	2.73E-06
ALDH1L1	5.397432603	4.384627635	-1.012804968	6.27E-08	1.85E-07
DNASE1L3	3.771218356	2.770189532	-1.001028824	4.42E-15	3.96E-14
PLK1	1.968202899	2.970361345	1.002158446	3.48E-34	5.49E-31
MARCKSL1	5.619876561	6.628641045	1.008764484	2.69E-16	3.02E-15
NEK2	2.196241596	3.206180484	1.009938889	1.54E-30	3.88E-28
PYCR1	2.607253856	3.617418619	1.010164763	6.41E-12	3.43E-11
SLC1A5	2.621423374	3.634463363	1.013039989	1.61E-16	1.89E-15
MCM3	4.676302582	5.68999691	1.013694328	9.36E-25	5.67E-23
DSG2	3.684885387	4.707392807	1.02250742	3.38E-13	2.19E-12
PBK	2.447284238	3.477476742	1.030192503	7.20E-25	4.42E-23
DTL	2.760357792	3.792852694	1.032494902	2.40E-25	1.61E-23
TACC3	2.841117511	3.882775171	1.04165766	5.63E-30	1.01E-27
KRT19	2.017821085	3.059891314	1.042070229	4.35E-06	9.81E-06
NCAPG	2.349244978	3.393011918	1.04376694	5.75E-30	1.01E-27
MELK	2.48997155	3.542010213	1.052038663	8.48E-28	8.80E-26
ASF1B	2.402744348	3.456452154	1.053707806	2.46E-30	5.47E-28
AURKB	2.29244832	3.347157729	1.054709409	1.60E-29	2.53E-27
BUB1B	2.265010191	3.320556143	1.055545951	9.25E-31	2.47E-28

LRRC1	2.263630893	3.324876939	1.061246046	1.49E-23	7.35E-22
AGR2	1.658240607	2.719600942	1.061360335	9.87E-06	2.12E-05
MCM6	4.05581915	5.123381455	1.067562304	2.14E-28	2.52E-26
CLGN	2.644766236	3.717585808	1.072819572	2.72E-13	1.80E-12
ECT2	2.50797978	3.5817695	1.07378972	1.21E-25	8.90E-24
S100P	3.587211443	4.663531004	1.076319561	3.24E-05	6.50E-05
MMP9	2.802090193	3.880411661	1.078321468	5.08E-12	2.75E-11
ASPM	2.842730722	3.922278653	1.079547931	1.53E-25	1.09E-23
CDCA8	2.430706882	3.527009316	1.096302435	7.55E-34	8.81E-31
KIF4A	2.308760705	3.40550007	1.096739365	3.98E-30	8.26E-28
CKS2	5.348296406	6.445682475	1.097386069	2.52E-23	1.20E-21
ROBO1	4.235273303	5.339493783	1.104220481	8.13E-13	4.97E-12
EPS8L3	2.644266399	3.750508951	1.106242552	3.21E-14	2.47E-13
KIF2C	2.180433496	3.293512347	1.113078851	2.98E-34	5.49E-31
FOXM1	2.434913092	3.548395525	1.113482433	1.59E-27	1.60E-25
LMNB1	2.786491656	3.915125349	1.128633692	1.68E-27	1.65E-25
PRC1	2.960891393	4.09514297	1.134251577	1.64E-27	1.63E-25
CENPM	2.423375295	3.559974866	1.136599571	7.21E-31	2.10E-28
NT5DC2	2.621914501	3.762134099	1.140219598	1.71E-20	4.36E-19
GAL3ST1	2.272862867	3.423229221	1.150366354	5.08E-15	4.50E-14
SFN	3.883254313	5.033945038	1.150690725	5.35E-08	1.60E-07
HMGA1	4.327884533	5.480155219	1.152270686	1.65E-29	2.53E-27
MDK	5.270362726	6.423948658	1.153585931	1.10E-10	4.86E-10
SOX4	2.903978019	4.064735273	1.160757254	1.44E-17	2.01E-16
UBD	6.714288095	7.879801788	1.165513692	3.28E-10	1.35E-09
UPK3A	1.933098835	3.106704833	1.173605998	6.88E-15	5.94E-14
KIF20A	2.376488081	3.553215303	1.176727222	8.07E-31	2.24E-28
NUSAP1	3.730995155	4.911049135	1.180053981	5.76E-25	3.64E-23
PAFAH1B3	3.358184638	4.542818844	1.184634205	8.80E-26	6.58E-24
VIL1	3.225555791	4.423603585	1.198047794	5.95E-12	3.19E-11
CCNB2	2.631204204	3.832135017	1.200930813	6.08E-31	1.83E-28
BAMBI	4.302118798	5.504158524	1.202039726	3.26E-16	3.60E-15
LAPTM4B	5.658808049	6.863196934	1.204388885	3.50E-18	5.72E-17
KIFC1	2.535601173	3.742636499	1.207035326	6.02E-34	8.04E-31
GINS1	2.749270202	3.964184783	1.214914582	1.78E-31	6.65E-29
MCM2	3.250800054	4.485768137	1.234968083	5.06E-28	5.56E-26
CDKN3	3.098653805	4.33937469	1.240720885	3.58E-26	2.83E-24
IGF2BP2	3.112044147	4.354966345	1.242922198	2.94E-17	3.90E-16
NDRG1	5.287002335	6.532905655	1.245903321	3.41E-21	1.03E-19
CCL20	4.314567325	5.571265926	1.256698602	8.17E-09	2.75E-08
RRM2	3.594488903	4.869471086	1.274982183	1.24E-25	9.02E-24
BIRC5	2.631656028	3.909987535	1.278331506	5.19E-31	1.73E-28

SPHK1	2.213209065	3.495626238	1.282417174	2.95E-20	7.19E-19
ZWINT	3.613711868	4.931329007	1.317617139	1.25E-30	3.25E-28
PTTG1	4.017021764	5.372458338	1.355436574	4.54E-29	6.15E-27
CA9	1.674178679	3.033554889	1.359376209	1.25E-20	3.28E-19
DUSP9	2.344854565	3.749920143	1.405065577	8.90E-17	1.09E-15
TPX2	3.420966296	4.833096601	1.412130305	5.72E-32	2.85E-29
G6PD	3.162521491	4.579357736	1.416836245	1.40E-25	1.00E-23
SPP1	5.92289037	7.340631904	1.417741534	3.47E-06	7.91E-06
SOX9	3.427991275	4.854268463	1.426277188	7.07E-19	1.33E-17
CCNB1	3.37725186	4.851511718	1.474259858	6.08E-33	4.37E-30
SPINK1	6.042303268	7.524688057	1.482384789	1.23E-05	2.61E-05
CD24	4.880487997	6.377235995	1.496747998	5.33E-12	2.88E-11
TOP2A	3.323142619	4.88099359	1.557850971	1.73E-29	2.60E-27
DKK1	2.080177484	3.654449883	1.574272399	1.18E-13	8.25E-13
MYBL2	2.584115958	4.227943491	1.643827533	4.93E-36	2.30E-32
CDC20	3.00914952	4.682372301	1.673222781	1.36E-33	1.27E-30
GPC3	6.73961691	8.438824148	1.699207238	4.03E-10	1.63E-09
PEG10	3.419129192	5.245290881	1.826161689	1.38E-10	5.97E-10
AFP	3.290265363	5.662138236	2.371872872	1.81E-12	1.05E-11

Supplementary Table 5: Differential genes between the low and high risk group.
